# Supplementary material for: Content-rich biological network constructed by mining PubMed abstracts
Source: BMC Bioinformatics. 2004 Oct 8;5:147. doi: 10.1186/1471-2105-5-147 (PMC528731; doi:10.1186/1471-2105-5-147)
Supplement: Additional File 2 — The original results of the above study (non-essential files are deleted to keep the file size under the limit set by BMC bioinformatics). [file 1471-2105-5-147-S2.bz2 › chilibotAdditionalFile2/dip40/573ID2164219E10940/html/CD28_CD80.html]

 


 **CD28** and **CD80** 
  
Found 551 abstracts in PubMed, retrieved 40.  
 

 What does Google say? 
 PDF only 
| .edu only 

---

**Interactive relationship** (e.g. stimulation, inhibition, etc)

**Stimulatory relationship**- **CD28**  binds to its ligand  **CD80**  or CD86 and transduces the most important costimulatory signal.  Ref: 12636255 Microbiol Immunol, 2003
- CD4 T cell activation is positively  **CD28**  and negatively CTLA 4 regulated by the costimulatory ligands  **CD80**  and CD86.  Ref: 12496387 J Immunol, 2003
- Many new vaccines will be based upon soluble recombinant antigens, and in vaccination with these antigens  **CD80**  and CD86 would normally be expressed on activated antigen presenting cells and additional stimulation through  **CD28**  would not be predicted to enhance responses further.  Ref: 12594842 Eur J Immunol, 2003
- Using chimeric rat mouse  **CD28**  molecules, we show that the superagonists bind exclusively to the laterally exposed C D loop of the immunoglobulin like domain of  **CD28**  whereas conventional, costimulatory mAbs recognize an epitope close to the binding site for the natural  **CD80**  CD86 ligands.  Ref: 12707299 J Exp Med, 2003
**Neutral relationship**- The interaction between  **CD28**  on T cells and  **CD80**  on APCs intensifies the linkage between TCR and MHC at the site of contact between T cells and APCs.  Ref: 12444120 J Immunol, 2002
- The importance of interactions of  **CD80**  CD86 and  **CD28**  in activating CD4 T cells was clear.  Ref: 12794123 J Immunol, 2003
- Blocking  **CD80**   **CD28**  or ICAM 1 LFA 1 interactions alters synapse morphology and reduces the area and density of accumulated complexes.  Ref: 12444111 J Immunol, 2002
- Cell surface interactions between the T cell costimulatory receptors,  **CD28**  and cytotoxic T lymphocyte antigen 4 CTLA4, with their cognate ligands,  **CD80**  and CD86, on antigen presenting cells play an important role in T cell activation.  Ref: 12429713 J Leukoc Biol, 2002
- Condensed aromatic peptide family of microbial metabolites, inhibitors of  **CD28**   **CD80**  interactions.  Ref: 12565974 Bioorg Med Chem Lett, 2003
- An effective humoral response requires bidirectional interactions between B cells and CD4 T cells, critical of which are interactions between  **CD80**  CD86 expressed on activated B cells and  **CD28**  expressed on responder CD4 T cells.  Ref: 12794123 J Immunol, 2003
- The  **CD28**  independence of effector T cells within germinal centers suggested that a key function for  **CD80**  CD86 under these circumstances might be to provide negative regulatory signals via the  **CD28**  homologue CTLA 4.  Ref: 12496387 J Immunol, 2003
- The CD4 T cells expressed the counter receptors  **CD28**  and CTLA 4, and bound with the BB 1  [ **CD80** ]  positive muscle fibers in cell to cell contact.  Ref: 12409186 J Neurol Sci, 2002
- Fas ligand FasL, that has the inherent capacities to tether the T cell inhibitor FasL CD95 ligand to the surfaces of B7  **CD80**  and CD86 positive APC via CTLA 4 B7 interaction, and in so doing, to simultaneously interfere with B7 to  **CD28**  T cell activation signals.  Ref: 12794109 J Immunol, 2003
- **CD28**  and CTLA 4 are opposing regulators of T cell activation, triggered by the two ligands  **CD80**  and CD86.  Ref: 12355442 Eur J Immunol, 2002
- BACKGROUND  **CD80**  and CD86, cell surface molecules found only on antigen presenting cells APCs, are required for activation of CD4 postitve CD4 T cells by interaction with  **CD28**  cytotoxic T lymphocyte associated antigen 4 on T cells.  Ref: 12722029 Am J Kidney Dis, 2003
- The CD86 major histocompatibility complex class II MHC II cells and  **CD28**  CD4 T cells were decreased by SST treatment, while  **CD80**  MHC II cells, CD40 MHC II cells and CD154 CD4 T cells showed no change.  Ref: 12501009 Jpn J Pharmacol, 2002
- Using soluble fusion proteins soluble CTLA 4,  **CD28** , and  **CD80**  to block either  **CD28**  on the surface of T cells or  **CD80**  on the surface of APCs, it was demonstrated that  **CD80**  acquisition by T cells is mediated through its receptors, possibly  **CD28**  interaction.  Ref: 12444120 J Immunol, 2002
- Attempts at blocking the  **CD28**  B7s  **CD80**  CD86 pathway are currently underway with the receptor fusion protein, LEA29Y a second generation CTL4Aig, and humanized mAbs to CD 80  [ **CD80** ]  and CD86.  Ref: 12773967 Minerva Urol Nefrol, 2003

**Non-interactive relationship** (e.g. studied together, co-existance, homology, etc.)

- These results demonstrate, for the first time, a critical contribution of the  **CD28**   **CD80**  CD86 costimulatory pathway not only to natural protection against primary T. cruzi infection but also to DNA vaccine induced protective immunity to Chagas disease.  Ref: 12761091 Infect Immun< MedlineTA>Infect Immun, 2003
- The  **CD80**  86  **CD28**  and CD40 CD40 ligand costimulatory pathways are essential for Th cell dependent B cell responses that generate high affinity, class switched Ab in vivo.  Ref: 12517941 J Immunol, 2003
- The  **CD28**   **CD80**  CD86 mediated T cell costimulatory pathway has been variably implicated in infectious immunity.  Ref: 12761091 Infect Immun< MedlineTA>Infect Immun, 2003
- the contribution of  **CD28**  B7 costimulation to allergen induced release of TLCA was determined through use of CTLA4 Ig fusion protein and neutralizing monoclonal antibodies to  **CD80**  B7.1 and CD86 B7.2.  Ref: 12464958 J Allergy Clin Immunol, 2002
- Expression of CD4, CD8alpha, CD40, CD86,  **CD28** , CD31 and vascular endothelial growth factor was not different between the two tumor groups but expression of CD40 ligand,  **CD80** , NK1.1 and CXCR3 was relatively lower in A11 Mig tumors.  Ref: 12792744 Oncol Rep< MedlineTA>Oncol Rep, 2002
- **CD80** , CD86,  **CD28**  and Cytotoxic T lymphocyte antigen 4 CTLA 4 are well known co stimulatory molecules that form the major co stimulatory pathway essential for full activation of T cells.  Ref: 12679556 J Vet Med Sci, 2003
- the ablation of a normal response following the addition of neutralizing anti CD86  **CD80**  Abs mirrored the response of CD4 T cells to B cells of HIV viremic patients, while the addition of exogenous  **CD28**  ligands partially restored the poor CD4 T cell response to the B cells of HIV viremic patients.  Ref: 12794123 J Immunol, 2003
- In this study, we investigated the role of this costimulatory pathway in resistance to Trypanosoma cruzi infection by using  **CD28**  deficient mice and blocking antibodies against  **CD80**  and CD86.  Ref: 12761091 Infect Immun< MedlineTA>Infect Immun, 2003
- In this study, we report the construction of a monovalent single chain Fv antibody fragment from a high affinity anti human  **CD28**  antibody  **CD28** .3 that blocked adhesion of T cells to cells expressing the  **CD28**  receptor  **CD80** .  Ref: 12649149 Blood< MedlineTA>Blood, 2003
- **CD28**  is an important costimulatory molecule for T cells, and it has been shown that cell surface expression of its ligands,  **CD80**  and CD86, can enhance cellular immune responses against tumor cells these tumor cells do not normally express the ligands.  Ref: 12594842 Eur J Immunol, 2003
- The result of the present study indicated the potential role of the  **CD28**   **CD80**  co stimulation pathway in pathogenesis of allergic diseases in dogs.  Ref: 12679556 J Vet Med Sci, 2003
- Melanoma patients with thinner tumors and those surviving revealed an increase of CD19  **CD80**  and CD19  **CD80**  CD86 cells, as well as a higher concentration of CD3 CD4  **CD28**  cells.  Ref: 12878358 Hum ImmunolHum Immunol, 2003
- Thus, while disrupting either the  **CD80**  86  **CD28**  or the CD40 CD40 ligand costimulatory pathway abrogates T dependent B cell immune responses, the two pathways are nonredundant and mediated by distinct mechanisms.  Ref: 12517941 J Immunol, 2003
- We determined the concentration of peripheral blood lymphocytes, which expressed the  **CD80**  CD86 or  **CD28**  CTLA 4 molecules, in 38 patients with cutaneous melanoma and 27 controls.  Ref: 12878358 Hum ImmunolHum Immunol, 2003
- Antibodies to the  **CD28**  counter ligands  **CD80**  and CD86 inhibited allergen induced release of TLCA in mild asthmatic explants by 94% P < ;.  Ref: 12464958 J Allergy Clin Immunol, 2002
- We investigated expression of costimulatory molecules BB 1  [ **CD80** ] , B7 1  **CD80** , B7 2 CD86, and their counter receptors  **CD28**  and CTLA 4 CD152 in muscle biopsy specimens of patients with scleroderma polymyositis overlap syndrome SSc PM, primary polymyositis PM, and other related diseases to examine whether the muscle fibers in patients with SSc PM behave as antigen presenting cells APCs.  Ref: 12409186 J Neurol Sci, 2002
- High expression of  **CD28** ,  **CD80** , CD86, CD40, CTLA4, CD44v variants, and FasL occurred in alopecia areata resistant mouse spleens.  Ref: 12485450 J Invest Dermatol, 2002
- The costimulatory pathway  **CD28**   **CD80**  CD86 has been shown to play an important role in T cell activation in allergic asthma, but less is known about the effect of other costimulatory molecules in allergy.  Ref: 12355440 Eur J Immunol, 2002
- Critical Contribution of  **CD28**   **CD80**  CD86 Costimulatory Pathway to Protection from Trypanosoma cruzi Infection.  Ref: 12761091 Infect Immun< MedlineTA>Infect Immun, 2002
- These include the costimulatory ligand receptor pairs, such as CD40 CD154 and  **CD80**  CD86  **CD28** , CD52 and CD45.  Ref: 12517277 Expert Opin Biol Ther, 2002
- Changes in the number of  **CD80** , CD86, and  **CD28**  peripheral blood lymphocytes have prognostic value in melanoma patients.  Ref: 12878358 Hum ImmunolHum Immunol, 2002
- We hypothesized that in relapsing remitting MS RRMS with or without of IFNbeta treatment and in secondary progressive patients SPMS IL12 and costimulatory molecules  **CD80**  B7 1, CD86 B7 2,  **CD28** , CD40, CD40L would be differentially produced or expressed by MO or T cells.  Ref: 12672403 Clin Immunol, 2003
- Subsequent CTL induction by activated DC requires  **CD80**  CD86 on the DC to interact with the  **CD28**  costimulatory receptor on CD8 T cells.  Ref: 12445291 Immunol Rev, 2002
- Additional phenotyping of these clones showed them to express CD25,  **CD28** ,  **CD80**  and 86 but not CD56 or 57.  Ref: 12709539 Rheumatology Oxford , 2003
- Expression of costimulatory molecules, CD49D and  **CD28**  on T cells and  **CD80**  and CD86 on monocytes, was unchanged.  Ref: 12753529 Respirology< MedlineTA>Respirology, 2003
- All these compounds exhibited good activity against  **CD28**   **CD80**  binding with an IC 50 of 0.4.38 and 0.22 microM, respectively.  Ref: 12565974 Bioorg Med Chem Lett, 2003
